# Supplementary material for: Conditional chemoconnectomics (cCCTomics) as a strategy for efficient and conditional targeting of chemical transmission
Source: eLife. 2024 Apr 30;12:RP91927. doi: 10.7554/eLife.91927 (PMC11060718; doi:10.7554/eLife.91927)
Supplement: Figure 3—source data 3. [file elife-91927-fig3-data3.zip › Figure 3 source data3 part 1.zip!/Figure 3-Source data 3-part1/1063-13-CRISPResso_Report_8cz_fsdr.zip!/CRISPResso_on_1063_15/1a.Read_barplot.pdf]

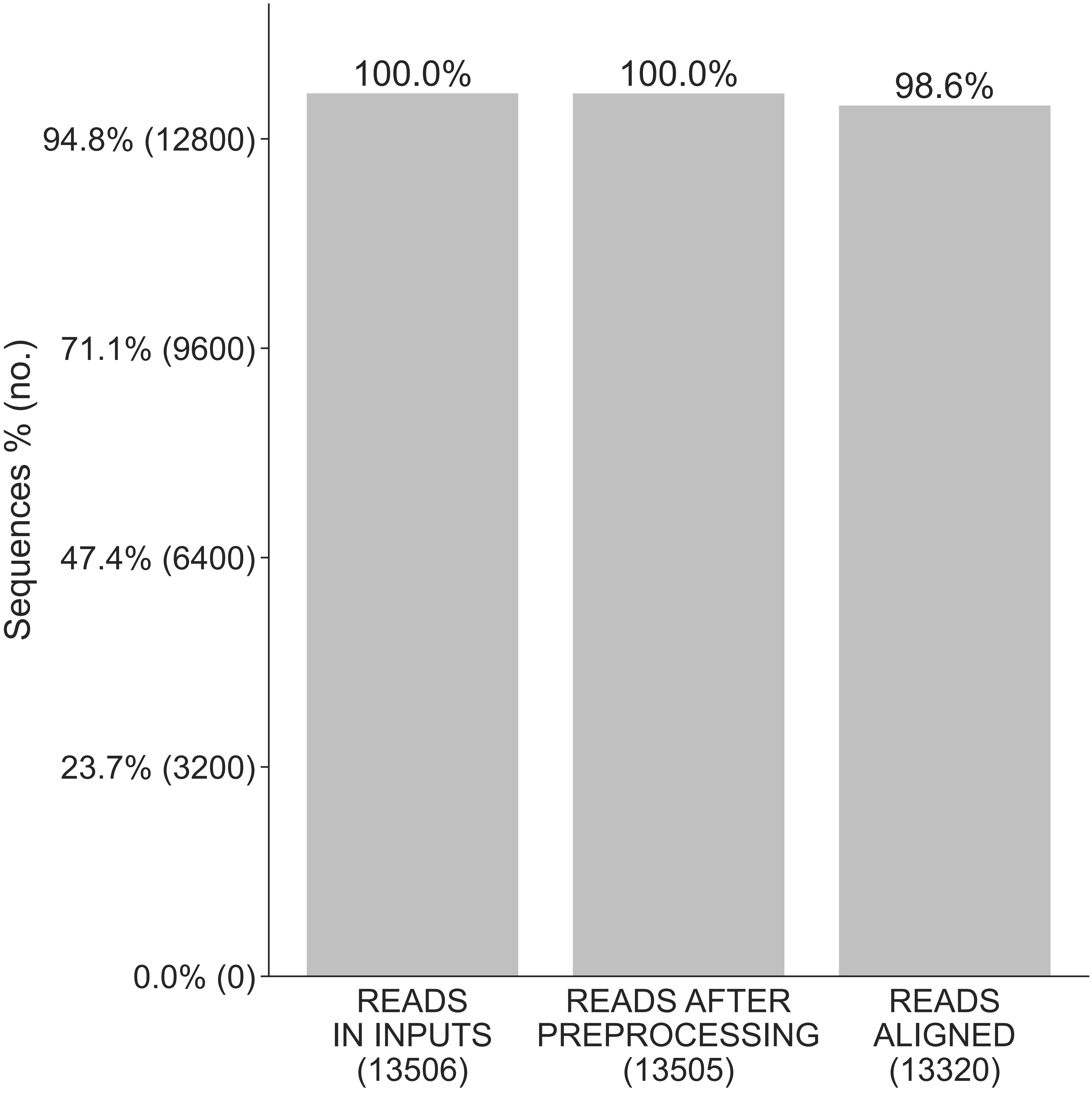
100.0%
100.0%
98.6%
94.8% (12800)
71.1% (9600)
47.4% (6400)
23.7% (3200)
0.0% (0)
Sequences % (no.)
READS IN INPUTS (13506)
READS AFTER PREPROCESSING (13505)
READS ALIGNED (13320)
